# Heteronormativity and the Justification of Gender Hierarchy: Investigating the Archival Data From 16 European Countries

**DOI:** 10.3389/fpsyg.2021.686974

**Published:** 2021-07-29

**Authors:** Federico Ferrari, Chiara Imperato, Tiziana Mancini

**Affiliations:** Department of Humanities, Social Sciences, and Cultural Industry, University of Parma, Parma, Italy

**Keywords:** heteronormativity, homonegativity, system justification, gender hierarchy, legitimizing myth

## Abstract

Within the framework of the System Justification Theory, this study tested on the archival data from 16 European countries the general hypothesis that homonegativity (HN), as an expression of gender binarism and heteronormativity, works as a legitimizing myth of gender hierarchy. Specifically, we hypothesized that (1) system justification (SJ) would positively relate to HN and (2) this relation would depend on the country level of gender hierarchy, (3) on the gender of respondents, and (4) on the interaction between gender hierarchy and gender. We selected the Gender Equality Index (GEI) as an indicator of the gender hierarchy of the country system and the items from the European Social Survey-Round 9 (ESS-9) as the indicators of the gender of respondents and the levels of SJ and HN. The Hierarchical Linear Modeling (HLM) partially confirmed the hypotheses, suggesting HN to work as a blatant prejudice and being more viable as a legitimizing myth in females from countries with higher gender hierarchy and in males from more gender-equal countries. In both cases, HN serves as a myth to justify the ontological premise of participants that the world is fair and to counteract the cognitive dissonance generated by the perception of a gender-unequal system (in the case of a woman) or by the perception of a gender-equal system that can threaten gender privileges (in the case of a man).

## Introduction

Within the Western value system, the principles of universalism and human rights have placed gender equality among the standards of a fair society, and the respect for sexual minorities has become one of the shared goals of the EU Institutions. Nonetheless, the complete achievement of such a goal appears to be still far from being realized, and in some European countries even more so. From the point of view of the rights of individuals and public policies, however, women equality and the social conditions of LGBT+ individuals (i.e., of Lesbian, Gay, Bisexual, Transgender, and other sexual minorities) are often the objects of separate actions, and the very definition of gender equality has often been cisgender-centric, that is to say strictly adherent to a definition of gender based on biological sex (Hines, 2007; Matthyse, 2020).

In recent times, this strategy appears to contradict the fact that Gender Theory has pointed out how the hierarchical and discriminatory gender system is based on heteronormativity as a common epistemology that belittles women as it marginalizes sexual minorities (Schilt and Westbrook, 2009; Ward and Schneider, 2009). Heteronormativity is defined as the belief that heterosexuality is the human default sexual orientation (Butler, 1990; Warner, 1991; Kitzinger, 2005; Habarth, 2015; Kowalsky and Scheitle, 2020). Heteronormativity implies a view of sexuality as strictly procreative and responding to a gender binary that aligns biological sex, gender identity, gender expression, gender roles, and sexual orientation, within a rigid, dichotomic, complementary, male–female gender schema (Bem, 1974, 1981). In other words, heteronormativity and gender binarism expect every individual to fall into either the masculine or the feminine category, which is clearly defined by the procreative physiology corresponding to his/her chromosomal sex. Such strict definition of sex would stem a natural sexual attraction to the opposite sex, as well as complementary attitudes and psychological traits; such gender roles would consider male individuals naturally dominant and aggressive and female individuals inherently submissive and nurturing (Eccles et al., 1990; Schilt and Westbrook, 2009). The psychosocial literature agrees that the above-described gender complementarity generates a power asymmetry, which favors men over women (Glick et al., 2000; Glick and Fiske, 2001). As an ideology that values certain social groups (i.e., either-male-or-female biologies, cisgender identities, heterosexual sexual orientation, and being a man) and stigmatizes others (i.e., intersex biologies, transgender identities, homosexual and bisexual sexual orientations, and being a woman), heteronormativity builds gender hierarchy and produces what has been called a “pyramid of sexual oppression” (Rubin, 1984; Halberstam, 1998).

Heteronormativity has been strictly related to sexism and sexual stigma and in particular to homonegativity (HN), which is defined as negative attitudes toward sexual minorities based on monitoring divergence from traditional masculine and feminine roles (Habarth, 2015; Scandurra et al., 2020). López-Sáez et al. (2020) found sexism and HN to be stronger, and resistance to heteronormativity to be lower, among groups higher in the gender hierarchy. As such, we can expect that gender binarism would not be enforced by those lower in the pyramid of sexual oppression and especially not by sexual minority populations. Nonetheless, some studies contradict this expectation by demonstrating that sexual minorities represent gender as a heteronormative male–female binary (Rocha Baptista and de Loureiro Himmel, 2016; Ferrari and Mancini, 2020; Kowalsky and Scheitle, 2020) and can also show high sexism and internalized HN (Tatum and Ross, 2020), especially when they adhere to conservative ideologies (López-Sáez et al., 2020). Thus, sexual minorities seem to express a sort of out-group favoritism that contradicts the Social Identity Theory (SIT; Tajfel and Turner, 1979) assumption that individuals would adhere to cognitions favorable to their own group.

While these findings can be explained as a case of consensual discrimination occurring when the intergroup status is perceived as stable and legitimate (Rubin and Hewstone, 2004), they may also be explicated by other psychosocial theories. For example, the Social Dominance Theory (SDT; Pratto et al., 2006) explains the out-group favoritism with the assumption that all social systems converge toward the formation of group-based stable social hierarchies, one of which is the male-dominated gender system based on gendered reproductive strategies. Moreover, gender would also influence individual differences in relation to his/her desire for hierarchically structured intergroup relations [i.e., social dominance orientation (SDO)], with men being inherently more oriented to it. However, the psychosocial theory that best sought to answer the question of the legitimation of group-based inequality at the expense of personal and group interest is the System Justification Theory (SJT; Jost and Banaji, 1994; Jost and Hunyady, 2002). SJT is the theory on which this study is focused.

System Justification Theory suggests that most people find ways of tolerating and justifying the group-based inequality as legitimate and necessary. To do so, they endorse minority-stigmatizing stereotypes, myths and ideologies legitimizing hierarchies, and out-group favoritism. SJT assumes that the out-group favoritism cannot be explained by theories stressing either *ego-justifying motives* to serve individual self-esteem or *group-justifying motives* to maintain or enhance collective self-esteem and/or positive group distinctiveness (for the contradictory view, see Owuamalam et al., 2019; Caricati and Owuamalam, 2020). SJT suggests the need for a *system-justifying motive* “to maintain or enhance the legitimacy and stability of existing forms of social arrangements” (Jost and Hunyady, 2002, p. 113). One of the predictions of SJT is that members of oppressed groups experience a stronger cognitive dissonance between system-justifying motive and ego- and group-justifying motives. To restore consonance, the disadvantaged would embrace stronger attitudes of justification of the status quo. However, this is not always or even ordinarily expected, especially when the system justification (SJ) conflicts with motives for self-enhancement, self-interest, and in-group favoritism (Jost et al., 2003).

The SJT has been widely applied to research on women and sexual minorities. For example, Bonnot and Krauth-Gruber (2017) found that women with a higher feeling of dependence on the social system remembered their own competencies as more similar to the gender stereotype. Moreover, SJ was widely used to explain why women held sexist behaviors and beliefs influencing their adhesion to political conservatism (Sibley et al., 2007; Russo et al., 2014; Hodson and MacInnis, 2017; Corrington and Hebl, 2018; Prusaczyk and Hodson, 2018; Cassese and Barnes, 2019), why they turned to more benign attributions for stranger harassment experiences (Saunders et al., 2017), rape myth acceptance, and rape victim blaming (Ståhl et al., 2010; Joseph et al., 2013; Chapleau and Oswald, 2014). SJT explained these effects with the “palliative function” of SJ that protects discriminated individuals from cognitive dissonance. Bahamondes et al. (2020) found SJ to have such a protective effect also on sexual minorities, among which the endorsement of system-justifying beliefs had a negative association with psychological distress, through a reduced perception of sexual discrimination. Research on SJ also widely tapped into ambivalent sexism findings (Napier et al., 2010) and, in particular, into the evidence that women were less likely than men to endorse a more hostile justification of gender inequality, but they still did justify it by leaning on the benevolent forms of sexism (Glick et al., 2000; Russo et al., 2014). In fact, despite important negative effects on the self-representation of women (Calogero and Jost, 2011), benevolent sexism is associated with greater life satisfaction, confirming the palliative effect hypothesized by the SJT (Connelly and Heesacker, 2012). Napier et al. (2010) found that whereas the ideology of benevolent sexism legitimized gender discrimination regardless of the level of national gender inequality, hostile sexism is related to life satisfaction only in gender-unequal nations. In other words, benevolent sexism as a myth legitimizing gender hierarchy did not depend on objective gender inequalities, while hostile sexism did.

All these results are consistent with the idea that heteronormativity, and its corollary benevolent justification of gender inequalities as “complementarity,” works as a legitimizing myth supporting the existing gender hierarchy. Therefore, heteronormativity can be regarded both as a myth legitimizing gender hierarchy and as one of the effects of the more general process of defending and justifying the status quo through stereotyping and ideological devices, according to SJT. Starting with the general hypothesis that HN, as an expression of heteronormativity, works as a legitimizing myth of gender hierarchy, this study aims at analyzing the relation between System Justification (SJ) and Homonegativity (HN) taking into account both the gender of respondents (as a personal factor) and the gender hierarchy/equality of the country as a societal factor as it was measured by the Gender Equality Index (GEI). Based on SJT, our first hypothesis was SJ to have a positive correlation with HN (Hypothesis 1). Second, in line with the results of the study by Napier et al. (2010), we expected this effect to depend on whether the system in the different countries would be more gender-hierarchical or more gender-equal. That is to say, we expected that the level of national gender hierarchy would moderate the relationship between SJ and HN, i.e., the positive correlation between SJ and HN would be higher in more gender-hierarchical countries than in more equal ones (Hypothesis 2). It would occur because members of gender-hierarchical countries need to endorse HN in order to align the awareness of living in a gender-hierarchical country and the belief in a justifiable system. Moreover, based on the prediction of SJT that members of oppressed groups embrace stronger attitudes of justification of the status quo to restore the experience of a stronger cognitive dissonance between system-justifying motive and ego- and group-justifying motives, we expected that the positive correlation between SJ and HN would be stronger among the female participants (Hypothesis 3). Finally, we also expected a national gender hierarchy × gender interaction predicting that the positive correlation between SJ and HN would be higher in females from more gender-hierarchical countries (Hypothesis 4).

## Methods

### Study Design and Procedure

This study is based on the archival data from 16 European countries. It aimed at comparing across the European countries at a micro-country or individual level (level 1) and at a macro-social or group level (level 2) the associations between the following factors:

- System justification, as the system-justifying stands of people (level 1),
- Heteronormativity, defined as the adhesion of people to gender binary ideology (level 1),
- Gender (level 1), and
- Gender hierarchy, indicated by the gender inequality of different countries (level 2).

To do so, it was decided at level 1 to draw from the microdata of European Social Survey-Round 9 (ESS-9; ESS Round 9, 2018) and at level 2 to draw from the archival data of the GEI (2019). Also, at level 2, we considered the data from Eurobarometer-493 (2019) and Eurobarometer 437 (2015) on acceptance of sexual diversity, World Bank Indicators (i.e., Ground Domestic Production and Gini Index), and personal sociodemographic information and personal values, using them as control variables.

The data were stored in an Excel matrix containing ESS-9 survey respondents in rows and selected items in columns. The level 2 indicators were placed in the same matrix, replicating them for each row referring to the country to which they belonged. The data matrix was then transferred to a secured OneDrive folder to which only the authors of this study had access.

### Measures

To test the hypotheses, we identified both design and covariate variables, and both individual-level societal-level indicators.

#### Individual-Level Indicators

For individual-level indicators, we considered the microdata from the ESS. The ESS is a biennial survey, collecting the cross-national data on attitudes and behaviors, in the cross-sectional probability samples, which are representative of all persons, aged 15 and above, resident in private households in each of the European participant countries. For this study, the data were used from ESS-9 (European Social Survey Round 9 Data, 2018) released in November 2019 and referring to 19 of the 27 countries surveyed in 2018 (*N* = 36,015; ESS-9 2018, edition 1.1. published on November 11, 2019). For each ESS-9 respondent, a few items were selected as indicators of the individual-level design variables.

##### System Justification

The research on SJT operationalized SJ in different ways as follows: (1) perceptions of fairness and legitimacy of the prevailing social system (Jost et al., 2003; Kay and Jost, 2003); (2) detection of a number of specific belief systems, such as Protestant Work Ethic, Belief in a Just World, Belief in Individual Mobility (O'Brien and Major, 2005), and political conservatism (Butz et al., 2017); and (3) trust and confidence in government, and empowerment of, and deference to authority (van der Toorn et al., 2011). This complicated the selection of specific indicators when working with the preexisting data of ESS-9, which did not include *ad hoc* measures, such as the System Justification Scale (Kay and Jost, 2003). Based on the various definitions of SJ, it was decided to select items from ESS-9 pertaining to three related dimensions (Table 1): the personal trust in and satisfaction with their country institutional system of the participants, and their belief in a just world. Through an interjudge agreement validation between the three authors, we selected four items from ESS-9 related to *trust in the system* (SJ_T; α = 0.90), five items related to *satisfaction for the system* (SJ_S; α = 0.82), and three items related to *belief in a just world* (SJ_J; α = 0.75).

**Table 1:** ESS-9 items used for system justification and homonegativity.

**ESS-9 items for system justification**	
SJ_T: Trust in the system	how much you personally trust each of the institutions I read out… …[country]'s parliament? …the legal system? …politicians? …political parties?
SJ_S: Satisfaction for the system	On the whole how satisfied are you with the present state of the economy in [country]? Now thinking about the [country] government, how satisfied are you with the way it is doing its job? And on the whole, how satisfied are you with the way democracy works in [country]? Now, …please say what you think overall about the state of education in [country] nowadays? …please say what you think overall about the state of health services in [country] nowadays?
SJ_J: Just world belief	I think that, by and large, people get what they deserve I am confident that justice always prevails over injustice I am convinced that in the long run people will be compensated for injustices
**ESS-9 items for homonegativity**	
Gay men and lesbians should be free to live their own life as they wish	
If a close family member was a gay man or a lesbian, I would feel ashamed	
Gay male and lesbian couples should have the same rights to adopt children as straight couples	

##### Homonegativity

As far as HN was concerned, we selected three items from ESS-9 (Table 1) referring to *attitudes toward Gay men and Lesbians* (i.e., the answer option was 1–5, 1 = agree strongly and 5 = disagree strongly) through an interjudge agreement validation. We calculated a synthetic indicator of HN (α = 0.80).

##### Gender

Information about gender was gained from ESS-9 item F2 asking about the sex of the respondent (i.e., the answer option was 1 = M, 2 = F, 9 = No answer). Answers were recoded into a dummy variable (Gender 1 = F).

##### Individual-Level Covariates

For each respondent of the ESS-9 database, we used the following *personal information* as control indicators: age, the highest level of education (recoded as a dichotomic response: Higher Education, i.e., 4–6 of the original questionnaire = 1), household income (1–9 decile), and bio-parental status (1 = had a child). Moreover, we drew items related to *basic human values* from the dedicated section of ESS-9. ESS-9 included 21 questions about the adhesion of respondents to Schwartz's values. The Schwartz Theory of Basic Human Values (Schwartz, 1992) maintains 10 transcultural human values grouped in 4 higher-order kinds of motivations. All questions were formulated asking the respondent to indicate how much he/she would feel to be like someone for whom some specific aspects of life are important. Answers were on a 7-point Likert scale, with 1 = “Very much like me” and 7 = “Not like me at all.” We formed four indexes, one for each Type of Motivation, calculating the average scores of all the items referring to each as follows: Openness to Change (α = 0.66), Self-enhancement (α = 0.72), Conservation (α = 0.70), and Self-transcendence (α = 0.74).

#### Group-Level Indicators

As the societal-level variables were concerned, indicators were chosen from different databases.

##### Gender Hierarchy, Equality

We considered the overall score of the 2019 GEI calculated from the indicators collected in 2017 about the actual situation of men and women in EU countries in six core domains as follows: work, money, knowledge, time, power, and health.

##### Group-Level Covariates

On a societal level, we decided to consider country indicators of acceptance of sexual diversity (as an index of the system cultural heteronormativity); economic inequality (Gini Index); and living standards and purchasing power parity (GDPppp). The level of *acceptance of sexual diversity* was measured using the data from Special Eurobarometer-493 (2019) and Special Eurobarometer 437 (2015) on discrimination, which included specific items on sexual discrimination. We decided to draw a synthetic index from the average scores of a few items that were identical in both 2015 and 2019 versions. In particular, both reports included the same six questions on attitudes toward gay, lesbian, bisexual, and transgender persons (e.g., “*From 1 to 10 how comfortable would you feel about having a gay, lesbian or bisexual person in the highest elected political position;” “From 1 to 10 how comfortable would you feel if a colleague at work with you were a transgender person;” “From 1 to 10 how comfortable would you feel if one of your children was in a love relationship with a person of the same sex”*) allowing to pair 2015 and 2019 data for each country, in order to identify a mean score referring to the climate of *Acceptance of Sexual Diversity* over the time period (ASD; α = 0.99). As a control indicator of the *economic status* of the examined countries, we retrieved the 2018 Gross Domestic Product (GDP) *per capita* from the World Bank database and converted it by using the Purchasing Power Parity (PPP) exchange rate in constant 2011 international dollar. This value reflects the average income in a country in relation to the cost of living. We also used the last available Gini Index (from 2017 in all cases, except for Germany, Great Britain, and Ireland, which were from 2016) from the World Bank database as a control indicator of the economic disparities within each country.

### Participants

Participants from countries not included in the GEI were excluded from the microdata ESS-9 2018 database. The analyzed sample was thus composed of 31,024 respondents from 16 countries as follows: Austria, Belgium, Bulgaria, Cyprus, the Czech Republic, Estonia, Finland, France, Germany, Great Britain, Hungary, Ireland, Italy, the Netherlands, Poland, and Slovenia.

The sample of respondents to ESS-9 was composed of 53.4% women, aged 15–90 years old (*M* = 50.86; *SD* = 18.73), 41.5% (*N* = 12.816) with a lower-tier education or less, and 58.5% (*N* = 18.039) with a higher education. As far as the marital status was concerned, 69.5% (*N* = 21.385) was or had been married or in a registered partnership and 69.9% (*N* = 21.613) had biological offspring. The household income was evenly distributed with 53.2% (*N* = 13.115) of respondents declaring to be on the 5th decile or less.

## Results

### Descriptive Statistics

Table 2 shows the descriptive statistics of the level 1 design variables for each of the 16 countries. Differences were found based on the country considered for SJ_T, *F*_(15)_ = 335.218, *p* < 0.001, η^2^ = 0.142; SJ_S, *F*_(15)_ = 501.366, *p* < 0.001, η^2^ = 0.198; and for SJ_J, *F*_(15)_ = 109.397, *p* < 0.001, η^2^ = 0.051 [λ = 0.71, *F*_(45, 90, 424)_ = 245.861, *p* < 0.001, η^2^ = 0.108]. Specifically, Bulgaria and Cyprus were at the lowest levels on all SJ dimensions, while Netherlands and Finland were at the highest levels of the same variables (*p* < 0.001). Differences were also found on HN, *F*_(15)_ = 794.181, *p* < 0.001, η^2^ = 0.282, showing that the countries at the lower levels on SJ dimensions reported the highest levels of heteronormativity.

**Table 2:** Descriptive statistics of level 1 design variables (*N* = 31.024).

**Country**	**SJ_T *M* (*SD*)**	**SJ_S *M* (*SD*)**	**SJ_J *M* (*SD*)**	**Gender (% of Women)**	**HN *M* (*SD*)**	**Open.** ***M* (*SD*)**	**Self-en.** ***M* (*SD*)**	**Cons.** ***M* (*SD*)**	**Self-tr.** ***M* (*SD*)**	**Age** ***M* (*SD*)**	**Hi. Ed.** ***M* (*SD*)**	**H. Inc.** ***M* (*SD*)**	**Bio-P.** ***M* (*SD*)**	***N***
Austria	5.12 (1.95)	6.44 (1.58)	3.21 (0.96)	53.86	2.22 (1.01)	5.07 (0.92)	5.01 (0.82)	5.46 (0.79)	6.01 (0.71)	51.56 (18.04)	0.31 (0.46)	4.96 (2.57)	0.69 (0.46)	2,499
Belgium	4.67 (1.91)	5.95 (1.45)	2.99 (0.80)	50.87	1.87 (0.89)	5.22 (0.78)	4.90 (0.69)	5.33 (0.68)	5.98 (0.55)	47.91 (19.18)	0.66 (0.47)	5.70 (2.46)	0.68 (0.47)	1,767
Bulgaria	2.48 (2.09)	3.43 (1.83)	2.81 (0.93)	55.59	3.35 (0.88)	4.49 (1.10)	4.60 (0.95)	5.42 (0.86)	5.46 (0.84)	54.55 (18.12)	0.70 (0.46)	4.24 (2.51)	0.81 (0.40)	2,198
Cyprus	3.45 (1.96)	4.32 (1.81)	2.79 (0.81)	53.13	3.14 (0.97)	5.09 (0.91)	4.65 (0.83)	5.79 (0.69)	6.18 (0.65)	54.44 (18.65)	0.67 (0.47)	4.60 (2.67)	0.78 (0.41)	781
Czech Republic	4.12 (2.15)	5.85 (1.58)	2.43 (0.87)	56.25	2.79 (0.91)	5.06 (0.88)	4.84 (0.90)	5.39 (0.77)	5.50 (0.75)	49.04 (17.56)	0.63 (0.48)	5.31 (2.78)	0.70 (0.46)	2,398
Estonia	4.61 (2.00)	5.49 (1.61)	3.02 (0.74)	56.03	3.01 (1.03)	4.86 (0.86)	4.35 (0.81)	5.16 (0.70)	5.96 (0.60)	50.73 (19.31)	0.81 (0.39)	5.60 (2.63)	0.76 (0.43)	1,904
Finland	5.75 (1.85)	6.64 (1.41)	2.96 (0.77)	51.68	2.03 (0.89)	5.11 (0.86)	4.39 (0.82)	5.19 (0.81)	6.11 (0.60)	50.90 (19.13)	0.81 (0.39)	6.07 (2.76)	0.69 (0.46)	1,755
France	4.01 (1.90)	4.57 (1.64)	2.74 (0.80)	54.58	1.82 (0.93)	4.92 (0.94)	4.36 (0.83)	5.09 (0.87)	5.95 (0.73)	52.37 (18.97)	0.53 (0.50)	4.99 (3.05)	0.74 (0.44)	2,010
Germany	4.80 (1.98)	5.57 (1.59)	3.04 (0.77)	48.60	1.93 (0.88)	5.08 (0.82)	4.60 (0.79)	5.14 (0.80)	6.08 (0.55)	49.65 (19.06)	0.50 (0.50)	6.07 (2.81)	0.65 (0.48)	2,358
Great Britain	4.22 (2.05)	4.93 (1.73)	2.85 (0.78)	54.42	1.87 (0.83)	5.11 (0.91)	4.45 (0.87)	5.23 (0.82)	5.97 (0.64)	52.40 (18.38)	0.64 (0.48)	5.21 (2.98)	0.71 (0.45)	2,204
Hungary	4.48 (2.23)	4.43 (2.21)	2.99 (0.96)	57.65	3.34 (1.01)	4.93 (0.90)	4.92 (0.90)	5.25 (0.75)	5.50 (0.79)	50.89 (18.47)	0.51 (0.50)	5.17 (2.74)	0.67 (0.47)	1,698
Ireland	4.42 (2.12)	5.32 (1.76)	2.91 (0.90)	52.39	1.91 (0.79)	5.11 (0.92)	4.54 (0.92)	5.41 (0.84)	5.96 (0.73)	52.23 (17.69)	0.66 (0.47)	4.60 (2.73)	0.69 (0.46)	2,216
Italy	3.93 (2.07)	5.01 (1.56)	3.11 (0.85)	52.71	2.61 (0.96)	4.85 (0.90)	4.80 (0.86)	5.61 (0.73)	5.69 (0.73)	51.28 (19.43)	0.46 (0.50)	4.78 (2.45)	0.61 (0.49)	2,745
Netherlands	5.84 (1.63)	6.39 (1.25)	3.04 (0.75)	50.20	1.59 (0.69)	5.23 (0.83)	4.77 (0.72)	5.08 (0.74)	5.92 (0.53)	48.66 (18.82)	0.48 (0.50)	6.56 (2.77)	0.65 (0.48)	1,673
Poland	3.61 (2.04)	5.23 (1.83)	3.11 (0.84)	52.66	3.18 (0.97)	4.76 (0.90)	4.52 (0.85)	5.48 (0.79)	5.67 (0.75)	47.62 (18.88)	0.51 (0.50)	5.38 (2.63)	0.69 (0.46)	1,500
Slovenia	3.23 (1.98)	4.82 (1.79)	2.79 (0.81)	53.71	2.69 (0.95)	5.40 (0.82)	5.13 (0.73)	5.78 (0.67)	6.11 (0.51)	49.35 (18.82)	0.61 (0.49)	5.33 (2.58)	0.47 (0.44)	1,318
Total *M* (*SD*)	4.33 (2.18)	5.31 (1.87)	2.93 (0.86)	53.00	2.41 (1.08)	5.01 (0.92)	4.67 (0.87)	5.35 (0.80)	5.84 (0.71)	50.86 (18.73)	0.58 (0.49)	5.30 (2.77)	0.70 (0.46)	

*SJ_T, system justification trust in the system; SJ_S, system justification satisfaction for the system; SJ_J, system justification belief in a just world; HN, homonegativity; Open., openness to change; Self-en., self-enhancement; Cons., Conservation; Self-tr., self-transcendence; Hi. Ed., Highest Education; H. Inc., Household Income; Bio-P., being a biological parent*.

Table 3 shows the descriptive statistics of the level 2 design variable for each of the 16 countries included.

**Table 3:** Descriptive statistics of level 2 design and covariate variables (*N* = 16).

**Country**	**GEI**	**ASD**	**Gini Ind**.	**GDPppp**
Austria	65.3	6.11	29.7	46.26
Belgium	71.1	7.29	27.4	43.582
Bulgaria	58.8	3.33	40.4	19.321
Cyprus	56.3	4.74	31.4	33.048
Czech Republic	55.7	5.05	24.9	33.436
Estonia	59.8	4.88	30.4	31.035
Finland	73.4	6.91	27.4	42.061
France	74.6	7.51	31.6	39.556
Germany	66.9	6.81	31.9	45.936
Great Britain	72.2	8.39	34.8	40.522
Hungary	51.9	4.88	30.6	28.465
Ireland	71.3	8.21	32.8	70.855
Italy	63	6.02	35.9	35.828
Netherlands	72.1	8.60	28.5	49.787
Poland	55.2	5.89	29.7	28.786
Slovenia	68.3	6.03	24.2	32.728
Total *M* (*SD*)	64.74 (7.54)	6.29 (1.49)	30.72 (4.08)	38.82 (11.67)

*GEI, Gender Equality Index; ASD, acceptance of sexual diversity; Gini Ind., Gini Index for economic inequality; GDPppp, living standards and purchasing power parity*.

The bivariate analysis found most correlations to be significant with *p* < 0.01, which is likely due to sample high numerosity. For this reason, to avoid the risk of overestimating relationships between the variables, we considered only correlations with *r* > 0.09 (Cohen, 1988). As far as the level 1 variables were concerned (Table 4), the bivariate correlation analysis found HN to have an intermediate correlation with older age (*r* = 0.226, *p* < 0.01), and a small one with having values motivated by conservation (*r* = 0.184, *p* < 0.01) and being a biological parent (*r* = 0.145, *p* < 0.01). HN also had a moderate negative correlation with values motivated by Openness to Change (*r* = −0.218, *p* < 0.01) and by Self-transcendence (*r* = −0.257, *p* < 0.01), and a small one with being part of a wealthier household (*r* = −0.168, *p* < 0.01), with having a higher education (*r* = −0.123, *p* < 0.01), and with two of the three dimensions of System Justification, i.e., SJ_T (*r* = −0.167, *p* < 0.01) and SJ_S (*r* = −0.118, *p* < 0.01). SJ dimensions showed no other significant correlations.

**Table 4:** Zero-order correlations at level 1 variables (*N* = 31.024).

	**SJ_T**	**SJ_S**	**SJ_J**	**HN**	**Gender**	**Open**.	**Self-en**.	**Cons**.	**Self-tr**.	**Age**	**Hi. Ed**.	**H. Inc**.	**Bio-P**.
**SJ_T**	1												
**SJ_S**	0.667^**^	1											
**SJ_J**	0.225^**^	0.244^**^	1										
**HN**	−0.167^**^	−0.118^**^	0.041^**^	1									
**Gender**	−0.021^**^	−0.044^**^	−0.027^**^	−0.075^**^	1								
**Open**.	0.044^**^	0.053^**^	0.067^**^	−0.218^**^	−0.074^**^	1							
**Self-en**.	0.048^**^	0.073^**^	0.141^**^	−0.036^**^	−0.103^**^	0.560^**^	1						
**Cons**.	−0.036^**^	0.025^**^	0.127^**^	0.184^**^	0.077^**^	0.033^**^	0.210^**^	1					
**Self-tr**.	0.062^**^	0.050^**^	0.031^**^	−0.257^**^	0.096^**^	0.348^**^	0.200^**^	0.434^**^	1				
**Age**	−0.062^**^	−0.073^**^	−0.044^**^	0.226^**^	0.033^**^	−0.283^**^	−0.296^**^	0.181^**^	−0.012^*^	1			
**Hi. Ed**.	0.126^**^	0.051^**^	−0.083^**^	−0.123^**^	0.012^*^	0.147^**^	0.060^**^	−0.093^**^	0.074^**^	−0.145^**^	1		
**H. Inc**.	0.168^**^	0.153^**^	0.019^**^	−0.168^**^	−0.111^**^	0.147^**^	0.150^**^	−0.125^**^	0.047^**^	−0.238^**^	0.287^**^	1	
**Bio-P**.	−0.071^**^	−0.066^**^	−0.034^**^	0.145^**^	0.100^**^	−0.186^**^	−0.181^**^	0.131^**^	0.010	0.505^**^	−0.037^**^	0.017^**^	1

* *p < 0.05*;

** *p < 0.01*.

*SJ_T, System Justification Trust; SJ_S, System Justification Satisfaction; SJ_J, System Justification Just World Belief; HN, homonegativity; Open., Openness to Change; Self-en., self-enhancement; Cons., Conservation; Self-tr., self-transcendence; Hi. Ed., Highest Education; H. Inc., Household Income; Bio-P., being a biological parent*.

Regarding the level 2 variables (Table 5), GEI had a very large correlation with ASD (*rho* = 0.878, *p* < 0.01) and with GDPppp (*rho* = 0.663, *p* < 0.01) and a small correlation with Gini Index (*rho* = 0.012, *p* < 0.05).

**Table 5:** Spearman's correlations at level 2 variables (*N* = 16).

	**GEI**	**ASD**	**Gini**	**GDP**
**GEI**	1			
**ASD**	0.878^**^	1		
**Gini**	0.012^*^	−0.048^**^	1	
**GDP**	0.663^**^	0.818^**^	−0.089^**^	1

** *p < 0.01*;

* *p < 0.05*.

*GEI, Gender Equality Index; ASD, acceptance of sexual diversity; Gini Ind., Gini Index for economic inequality; GDP, living standards and purchasing power parity*.

### Testing the Hypotheses

To test our hypothesis, we decided to run three nested two-level hierarchical models with random intercept and slopes, testing the main, the two-way, and the three-way interaction effects of the three dimensions of SJ (i.e., SJ_T, SJ_S, and SJ_J), GEI, and Gender on HN as a dependent variable. It was decided to run a hierarchical model targeting the design variables with their main interactions and all main effects of level 1 and level 2 covariates (Table 6; see also Supplementary Materials for slopes of significant interactions).

**Table 6:** Hierarchical linear model, estimates of fixed effects.

	**Parameter**	**Estimate**	***SE***	**Sign**.	**95% CI**	
					**Lower bound**	**Upper bound**
**Trust**						
	Intercept	2.572	0.050	<0.001	2.463	2.682
Design variables	SJ_T	−0.034	0.004	<0.001	−0.041	−0.026
	GEI	−0.030	0.012	0.028	−0.057	−0.004
	Gender (1 = female)	−0.215	0.011	<0.001	−0.237	−0.193
	SJ_T * GEI	−0.000	0.001	0.482	−0.001	0.001
	SJ_T * Gender	0.025	0.005	<0.001	0.016	0.035
	GEI * Gender	−0.003	0.002	0.100	−0.006	0.001
	SJ_T * GEI * Gender	0.001	0.001	0.383	−0.001	0.002
Covariates	Age	0.008	0.000	<0.001	0.007	0.009
	Household Income	−0.022	0.002	<0.001	−0.026	−0.017
	High Education	−0.149	0.012	<0.001	−0.173	−0.126
	Openness to change	−0.062	0.008	<0.001	−0.077	−0.047
	Self-enhancement	0.053	0.008	<0.001	0.037	0.070
	Conservation	0.255	0.008	<0.001	0.239	0.271
	Self-transcendence	−0.334	0.010	<0.001	−0.354	−0.315
	Biological parent	0.084	0.014	<0.001	0.057	0.112
	Gini	−0.005	0.013	0.691	−0.033	0.023
	GDP	−0.008	0.007	0.274	−0.023	0.007
	ASD	−0.138	0.075	0.093	−0.302	0.027
**Satisfaction**						
	Intercept	2.573	0.052	<0.001	2.461	2.685
Design variables	SJ_S	−0.008	0.005	0.075	−0.0172	0.001
	GEI	−0.031	0.012	0.029	−0.058	−0.004
	Gender (1 = female)	−0.214	0.011	<0.001	−0.236	−0.192
	SJ_S * GEI	−0.001	0.001	0.145	−0.002	0.000
	SJ_S * Gender	0.011	0.006	0.074	−0.001	0.022
	GEI * Gender	−0.002	0.002	0.338	−0.005	0.002
	SJ_S * GEI * Gender	0.002	0.001	0.057	−0.000	0.003
Covariates	Age	0.008	0.000	<0.001	0.007	0.009
	Household Income	−0.023	0.002	<0.001	−0.027	−0.019
	High Education	−0.156	0.012	<0.001	−0.180	−0.132
	Openness to change	−0.060	0.008	<0.001	−0.075	−0.044
	Self-enhancement	0.052	0.008	<0.001	0.035	0.068
	Conservation	0.254	0.008	<0.001	0.238	0.271
	Self-transcendence	−0.334	0.010	<0.001	−0.353	−0.315
	Biological parent	0.089	0.014	<0.001	0.062	0.116
	Gini	−0.004	0.013	0.741	−0.033	0.024
	GDP	−0.008	0.007	0.260	−0.024	0.007
	ASD	−0.142	0.077	0.091	−0.311	0.027
**Belief in just world**						
	Intercept	2.566	0.051	<0.001	2.456	2.676
Design variables	SJ_J	0.015	0.009	0.116	−0.034	0.003
	GEI	−0.030	0.012	0.028	−0.057	−0.004
	Gender (1 = female)	−0.208	0.011	<0.001	−0.230	−0.187
	SJ_J * GEI	0.003	0.001	0.011	0.001	0.006
	SJ_J * Gender	0.043	0.013	0.001	0.019	0.068
	GEI * Gender	−0.002	0.002	0.226	−0.005	0.001
	SJ_J * GEI * Gender	−0.009	0.002	<0.001	−0.013	−0.006
Covariates	Age	0.008	0.000	<0.001	0.007	0.009
	Household Income	−0.024	0.002	<0.001	−0.028	−0.019
	High Education	−0.152	0.012	<0.001	−0.176	−0.128
	Openness to change	−0.059	0.008	<0.001	−0.074	−0.043
	Self-enhancement	0.047	0.008	<0.001	0.031	0.063
	Conservation	0.247	0.008	<0.001	0.231	0.264
	Self-transcendence	−0.331	0.010	<0.001	−0.350	−0.311
	Biological parent	0.090	0.014	<0.001	0.063	0.117
	Gini	−0.005	0.013	0.721	−0.033	0.023
	GDP	−0.008	0.007	0.252	−0.024	0.007
	ASD	−0.145	0.076	0.082	−0.311	0.022

*GEI, Gender Equality Index; SJ_T, System Justification Trust; SJ_S, System Justification Satisfaction; SJ_J, System Justification Just World Belief; HN, Homonegativity; Gini, Gini Index for economic inequality; GDP, living standards and purchasing power parity; ASD, acceptance of sexual diversity. Dependent variable: HN. Principal effects and interactions for design variables and principal effects for Level-1 and Level-2 variables (N = 23871)*.

As far as Hypothesis 1 was concerned, i.e., the positive relationship between SJ dimensions and HN, the results showed a significant negative correlation between SJ_T and HN, while no relationships were found regarding SJ_S and SJ_J, thus partially contradicting our H1.

Regarding Hypothesis 2, i.e., the role of GEI in the SJ–HN relation, the results showed no significant interaction with either SJ_T or SJ_S. Nevertheless, SJ_J showed a significant interaction with GEI indicating that individuals from more gender-equal countries (i.e., with high GEI levels) showed higher HN when they believed in a just world (*t* = 2.929, *p* < 0.01); instead, the slope was not significant for a low level of GEI, thus not confirming H2.

Furthermore, with respect to Hypothesis 3, SJ_T showed a significant interaction with Gender indicating that HN decreased with the increase of the trust in the system in both genders (*t* = −2.159, *p* < 0.05), but primarily in males (*t* = −8.651, *p* < 0.001), thus contradicting our H3 for SJ_T dimension. No interactions were found regarding SJ_S. However, SJ_J showed a significant interaction with Gender, indicating that females showed significantly higher levels of HN when they had high SJ_J values (*t* = 6.642, *p* < 0.001), thus confirming H3 for the SJ_J dimension.

Finally, considering Hypothesis 4, no significant interactions of GEI or of Gender were found on SJ_T and SJ_S dimensions. Nevertheless, SJ_J showed a significant interaction with both GEI and Gender, indicating that among individuals from more gender-hierarchical countries (with low GEI), females showed higher levels of HN when they believed in a just world (*t* = 8.621, *p* < 0.001), as we hypothesized (H4). Interestingly, among individuals from more gender-equal countries (with high GEI), males showed higher levels of HN when they believed in a just world (*t* = 2.929, *p* < 0.01).

General results showed HN decreased in women and individuals of countries with higher gender equality in all the three hierarchical models. Furthermore, in all the three models, all level 1 covariates (e.g., age, household income, high education, openness to change, self-enhancement, conservation, self-transcendence, and bio-parental status) had significant effects on HN. Specifically, age, self-enhancement, conservation, and bio-parental status positively correlated with HN, while household income, high education, openness to change, and self-transcendence had negative correlations with HN. Regarding level 2 covariates (e.g., Gini, GDP, and ASD), they did not relate to HN.

## Discussion

This study aimed at applying SJT in justifying gender hierarchy in 16 European countries, analyzing the microdata from ESS-9 and the levels of gender equality in these countries. As for system justification, we considered three correlated dimensions as follows: personal trust in and satisfaction with the institutional system of the country, and belief in a just world. We hypothesized HN, as an expression of gender binarism and heteronormativity, to work as a legitimizing myth of gender hierarchy especially among participants from more gender-hierarchical countries and among women. Contradictorily, the results show no positive correlation between any of the indicators of system justification considered and HN. Nonetheless, the data confirmed women not trusting the system to have higher HN, in particular, in more gender-hierarchical countries.

The negative relationship between trust in the system and HN as well as the absence of a significant relationship between both satisfaction with the system and belief in a just world and HN were the unexpected results. Nonetheless, they could be ascribed to the possibility that gender inequality is not perceived as a core element of the institutional systems of the European countries. In fact, European countries declare to pursue gender equality as a shared value, as evidenced by institutional statements and international agreements on the subject. However, gender inequality is endemic in the countries considered, as evidenced by GEI scores ranging from 51.9 to 72.2 out of 100 with no country in the sample even close to reaching complete gender equality. The low score of HN among respondents with high levels of trust in an albeit gender-unequal system could therefore suggest the denial of injustice not to occur in reason of the status quo of the system, but in reason of hope for change, consistently with the Social Identity Model of System Attitudes (SIMSA) (Caricati and Owuamalam, 2020). Alternatively, similarly to the study of Bahamondes et al. (2020), we could ascribe it to a phenomenon of reduced perception of discrimination that allows those who trust the system to legitimize it. This would somehow resort to an “evasiveness” toward sexual diversity (López-Sáez et al., 2021): outward neutrality or acceptance of LGBT+ individuals without acknowledging their experience of disparities (Brownfield et al., 2018). Such deliberate choice to “not know” denying the violence suffered by sexual minorities (Cowan et al., 2005) would suggest a more subtle form of discrimination toward LGBT+, which does not lean on the homonegative ideology to legitimize gender hierarchy.

Coherently to these interpretations, and contrary to our Hypothesis 1, the results suggest that HN, i.e., negative attitudes toward LGBT people, is not among the aspects that individuals justify when they declare trust and satisfaction for the institutional and economic status quo of the system in which they live. Similarly, according to the HLM results, gender significantly moderates trust in the system—heteronormativity relation: contrary to what we expected, slopes indicated a lower HN with higher levels of trust in the system both in males and in females, but with a much steeper slope among men. We can argue that this result is coherent with the idea of men aligning their personal level of HN with the standards of the institutions they trust, so to defend the status quo through the justificatory idea that actual discrimination is low, possibly leaning on evasiveness toward LGBT+ discrimination (Brownfield et al., 2018). The same possibility is less viable for women: on one hand, unlike men, women do not use HN to justify gender privilege, and, on the other hand, they are the ones directly faced with discrimination.

However, we found a different and more nuanced pattern in relation to belief in a just world, which we identified as the core indicator of justification of the system. The bivariate correlations found only a small negative association between belief in a just world and HN, and HLM indicated belief in a just world to have no main effect on HN. Nevertheless, the HLM results showed women to embrace more homonegative attitudes when they believed in a just world, although being a group directly concerned by gender discrimination. Coherently with the study of Napier et al. (2010), this effect was stronger in more gender-hierarchical countries where oppression on women is heavier, and women seemed to lean more on homonegative beliefs to justify their condition. The same occurred for men from more gender-equal countries, who were also more homonegative when they believed in a just world. We can therefore speculate that, on the one hand, women as a still discriminated group find themselves in cognitive dissonance believing in a just world, especially when they live in countries where gender discrimination is stronger. On the other hand, men, coherently with the idea of using HN as a legitimizing myth of their privilege, hold stronger homonegative attitudes when they live in gender-hierarchical countries but when they live in more gender equal countries they hold stronger homonegative attitudes if they believe in a just world.

Moreover, trust in and satisfaction with the system are also intuitively associated with the general well-being of the country, and more gender-equal countries are also the wealthier ones and those with a lower wealth disparity, as it is suggested by the positive correlation between GEI and GDPppp. The inverse correlation between Gini Index and GEI suggests that it is possible to ascribe the lack of interaction of SJ_T and SJ_S with GEI to some coherence in their relationship with GDPppp, which would align also their effects on HN. This would mean that the relationships between GEI and HN and between GDPppp and SJ across the countries would have the same direction, resulting in the lack of significance of the interaction between GEI and SJ on HN. Therefore, any significance (or lack of) of the interaction between GEI and SJ_T as well as between GEI and SJ_S should be taken cautiously, although not supporting the hypotheses H1 and H2 of HN working as a legitimizing myth of gender hierarchy.

Finally, the results confirmed HN as an attitude coherent with traditional views, as positive correlations of the design variables with age, cultural and economic status, and conservative values showed. The fact that gender hierarchy strongly associates with discrimination against LGBT+ individuals at the country level suggests HN as a good indicator of heteronormativity intended as an ideology underpinning both gender inequality and discrimination against LGBT+ individuals (Kowalsky and Scheitle, 2020). Moreover, coherently to the concept of heteronormativity and sexism being a whole belief system that regulates both the male–female relationships and the attitudes toward gender minorities, the results of this study showed that living in countries with more equal relationships between men and women, as well as belonging to a gender-oppressed group not directly affected by anti-LGBT attitudes, is associated with lower HN. In this sense, the tendency of women to be less homonegative than men could be considered coherent with the refusal of an ideology that indirectly penalizes all the social groups that are lower in the pyramid of sexual oppression, as it would be expected from the SIT (Tajfel and Turner, 1979).

## Conclusion

This study showed the indicators of system justification considered (i.e., personal trust in and satisfaction with the legal system of the country, and belief in a just world) to behave differently. The results suggested that belief in a just world is the more convincing justifying motive for endorsing HN, first of all in females coming from more hierarchical countries. Thus, the results suggest the opportunity to consider a more complex frame, in which different indicators of system justification behave differently for men and women, in justifying gender hierarchy in more gender-equal countries vs. more gender-hierarchical ones. Synthesizing, the relationships between different system justification measures and HN suggest taking into consideration two elements as follows: the social positioning of different gender subjectivities; and the contradiction of EU between the institutional engagement for gender equality and the still gender-hierarchical status quo.

Our results suggest that within the articulated ideology of gender binarism and heteronormativity, HN—along with hostile sexism—should be considered as a blatant prejudice, and this may condition the way HN can work as a legitimizing myth of gender hierarchy, being less viable for oppressed gender groups and in contexts where the political goal is gender equality. Nonetheless, HN works according to the prediction of SJT of a palliative function to restore cognitive consonance among oppressed groups, i.e., in the case of women from more gender-hierarchical countries. To believe in the fairness of a system that considers gender equality fundamental but fails to concretize it, women may lean upon the idea that heteronormativity is acceptable, and HN may express this legitimization. In this case, heteronormativity rather than justifying a system intended as a “State,” or a specific “organization of rights and laws,” seems to be useful for justifying one's own ontological premises in the world: the need to see the world as equitable means also to accept heteronormativity. This was true not only for the oppressed groups (in the case of women), especially in those more gender-hierarchical countries where there seems to be less alternative to the status quo, but also for the dominant group of men, at least in those more gender-equal countries where the societal norms force them to give up their privilege.

### Limitations

Most of the limitations of the study are related to the use of the archival data and the necessity to lean on the predefined items that were not conceived for our hypothesis. For instance, gender was a dichotomous variable, and it was not possible to distinguish sexual minorities among the respondents. Moreover, the indicators of system justification related to trust in and satisfaction with the political and institutional system might fail to detect the justification of respondents of the specific gender system. This might occur according to the hypothesis by Sengupta et al. (2015) that SJ works differently for specific aspects of social systems: in this case, asking about the generic belief of respondents about the institutions of their country (i.e., government, health, and education) might fail in detecting their attitudes toward the gender system.

Considering the limitations of this study, and some weaknesses in measures and statistical indexes (e.g., Cronbach's alpha or the Gini Index not being equal for each country), other dedicated studies could be conducted to further test the results we obtained. Moreover, future guidelines would use transnegativity as an indicator of heteronormativity to investigate the legitimization of gender hierarchy, since transgender subjectivities represent a more direct and socially pathologized break of heteronormativity than homosexuality does; hence, transnegativity may be more tolerated by the societal norms, working as a less blatant prejudice.

## Data Availability Statement

Publicly available datasets were analyzed in this study. This data can be found here: https://www.europeansocialsurvey.org/data/download.html?r=9 https://data.europa.eu/euodp/en/data/dataset/S2251_91_4_493_ENG https://data.europa.eu/euodp/it/data/dataset/S2077_83_4_437_ENG.

## Ethics Statement

Ethical review and approval was not required for the study on human participants in accordance with the local legislation and institutional requirements. Written informed consent for participation was not required for this study in accordance with the national legislation and the institutional requirements.

## Author Contributions

FF and TM contributed to conception and design of the study. FF organized the database and wrote the first draft of the manuscript. FF and CI performed the statistical analysis. CI wrote sections of the manuscript. All authors contributed to manuscript revision, read, and approved the submitted version.

## Conflict of Interest

The authors declare that the research was conducted in the absence of any commercial or financial relationships that could be construed as a potential conflict of interest.

## Publisher's Note

All claims expressed in this article are solely those of the authors and do not necessarily represent those of their affiliated organizations, or those of the publisher, the editors and the reviewers. Any product that may be evaluated in this article, or claim that may be made by its manufacturer, is not guaranteed or endorsed by the publisher.
