# Supplementary material for: Heteronormativity and the Justification of Gender Hierarchy: Investigating the Archival Data From 16 European Countries
Source: Front Psychol. 2021 Jul 29;12:686974. doi: 10.3389/fpsyg.2021.686974 (PMC8359921; doi:10.3389/fpsyg.2021.686974)
Supplement: Supplementary file 1 [file Table_1.DOCX]

**Supplementary material**. Slopes of significant interactions.
